# Analysis of a Lipid/Polymer Membrane for Bitterness Sensing with a Preconditioning Process

**DOI:** 10.3390/s150922439

**Published:** 2015-09-04

**Authors:** Rui Yatabe, Junpei Noda, Yusuke Tahara, Yoshinobu Naito, Hidekazu Ikezaki, Kiyoshi Toko

**Affiliations:** 1 Research and Development Center for Taste and Odor Sensing, Kyushu University, Nishi-ku, Fukuoka 819-0395, Japan; 2 Graduate School of Information Science and Electrical Engineering, Kyushu University, Nishi-ku, Fukuoka 819-0395, Japan; E-Mails: j.noda@nbelab.ed.kyushu-u.ac.jp (J.N.); tahara@belab.ed.kyushu-u.ac.jp (Y.T.); toko@ed.kyushu-u.ac.jp (K.T.); 3 Intelligent Sensor Technology, Inc., 5-1-1 Onna, Atsugi-shi, Kanagawa 243-0032, Japan; E-Mails: naitou.g@insent.co.jp (Y.N.); Ikezaki.Hidekazu@insent.co.jp (H.I.)

**Keywords:** taste sensor, lipid, surface, FTIR, XPS, GCIB-TOF-SIMS

## Abstract

It is possible to evaluate the taste of foods or medicines using a taste sensor. The taste sensor converts information on taste into an electrical signal using several lipid/polymer membranes. A lipid/polymer membrane for bitterness sensing can evaluate aftertaste after immersion in monosodium glutamate (MSG), which is called “preconditioning”. However, we have not yet analyzed the change in the surface structure of the membrane as a result of preconditioning. Thus, we analyzed the change in the surface by performing contact angle and surface zeta potential measurements, Fourier transform infrared spectroscopy (FTIR), X-ray photon spectroscopy (XPS) and gas cluster ion beam time-of-flight secondary ion mass spectrometry (GCIB-TOF-SIMS). After preconditioning, the concentrations of MSG and tetradodecylammonium bromide (TDAB), contained in the lipid membrane were found to be higher in the surface region than in the bulk region. The effect of preconditioning was revealed by the above analysis methods.

## 1. Introduction

Various types of sensor for mimicking the five human senses have been realized. These sensors are categorized into physical and chemical sensors. For example, imaging devices, microphones, and pressure sensors are categorized as physical sensors corresponding to the visual sense, auditory sense and tactile sense, respectively. In particular, rapid advances have been achieved in the performance of imaging devices, which has had an impact on society because imaging devices with high performance are readily available at a low cost. On the other hand, there are few commercialized chemical sensors. There are a number of methods for measuring chemical substances such as gas chromatography mass spectrometry (GCMS), liquid chromatography mass spectrometry (LCMS), high performance liquid chromatography (HPLC), FTIR and nuclear magnetic resonance (NMR). However, these methods should be carried out by specialists with high technical knowledge because the obtained data are complicated, whereas it is desirable for users to be able to easily discriminate and quantify chemical substances using a sensor device.

A taste sensor, a kind of electronic tongue, has been developed and commercialized as a chemical sensor to evaluate the taste of substances [1,2,3,4,5,6,7,8,9,10,11,12,13,14]. It is possible to measure the taste of foods or medicines using the taste sensor, which mimics the taste sense of humans, which is categorized into six types of taste; saltiness, sourness, umami, bitterness, astringency and sweetness. The taste sensor has global selectivity, which means that each of its membranes respond to substances that are categorized in each tastes. Several types of lipid/polymer membrane are used for the taste sensor, which consist of a polymer, plasticizer and lipid. The polymer is used to support the membrane and the plasticizer and lipid are used to control the hydrophobicity and electrical properties of the membrane, respectively. Then, the lipid/polymer membrane transduces the response to a taste into an electrical signal [6].

The measurements performed by the taste sensor are carried out using several types of lipid/polymer membrane designed to detect saltiness, sourness, umami, bitterness, astringency and sweetness. Two types of value are obtained from the measurements. First, a relative value is obtained by immersing the sensor electrodes in a sample solution. Second, a change in the membrane potential caused by adsorption (CPA) value is obtained by immersing the sensor electrodes in another standard solution after the measurement of the sample solution. The two values are used to evaluate the initial taste and aftertaste. The CPA can be obtained using lipid/polymer membranes for umami, bitterness and astringency. It is known that the CPA is related to the adsorption of taste substances on the membrane [15,16,17]. In this study, we focused on the lipid/polymer membrane for sensing bitterness, which is called “C00”. It was previously reported that the CPA became stable after the C00 membrane was immersed in monosodium glutamate (MSG) solution for several days [18]. In this study, we analyzed the change in the surface structure of the lipid/polymer membrane for bitterness sensing by immersing it in MSG solution.

## 2. Experimental Section 

### 2.1. Materials

Polyvinyl chloride (n about 1100, PVC) was purchased from Wako Pure Chemical Industry (Osaka, Japan) as a polymer material. 2-Nitrophenyl octyl ether (NPOE) was obtained from Dojindo Laboratories (Kumamoto, Japan) as a plasticizer. Tetradodecylammonium bromide (TDAB) was purchased from Sigma Aldrich (St. Louis, MO, USA) as a lipid reagent. Tetrahydrofuran (THF) was obtained from Sigma Aldrich as a solvent. Sodium hydrogen L-glutamate monohydrate (monosodium glutamate, MSG), potassium chloride (KCl) and L(+)-tartaric acid were purchased from Kanto Chemical, Co., Inc. (Tokyo, Japan). Iso-alpha acid was obtained from Intelligent Sensor Technology Inc. (Kanagawa, Japan). Hydrogen peroxide (30%, Wako Pure Chemical Industry) and sulfuric acid (Kanto Chemical, Co., Inc.) were purchased for use as piranha solution. The standard solution in this study was made from 30 mM KCl and 0.3 mM tartaric acid in water. All aqueous solutions were prepared from Milli-Q water obtained from a Milli-Q system (Millipore, Billerica, MA, USA).

### 2.2. Fabrication of Lipid/Polymer Membrane for Bitterness Sensing 

The lipid/polymer membrane for bitterness sensing was fabricated by the following process: first, TDAB was dissolved in THF. Next, NPOE and the TDAB solution were mixed. Then, PVC was added to the mixed solution. After that, the mixed solution was dried on a Petri dish at room temperature for 3 days to obtain a membrane sample. Next, the membrane sample was immersed in MSG solution (30 mM MSG in standard solution) at room temperature for about 7 days (the MSG solution should be remade each day because bacteria breed in the solution). Finally, the membrane sample was cut and attached to the sensor surface using PVC in THF solution to form a sensor electrode. This fabrication process was used in our previous study [6]. 

### 2.3. Measurement Procedure of Taste Sensor 

The relative value and CPA value were measured using a taste sensor system (TS-5000Z, Intelligent Sensor Technology, Inc.). Several electrodes were prepared for the taste sensor system. One of them was a reference electrode, which was a Ag/AgCl electrode immersed in saturated KCl solution. The others were sensor electrodes that were made from the lipid/polymer membrane. The potentials of these electrodes were generated by immersing them in a sample solution. The output was the electrical potential difference between the sensor electrode and the reference electrode.

The measurement procedure was carried out by the following steps. First, the electrodes were immersed in the standard solution and the sensor output was measured as *V_r_*. Next, the electrodes were immersed in the sample solution and the output was measured as *V_s_*. Then, the electrodes were immersed in another standard solution and the output was measured as *V_r_’*. The relative value and CPA value were calculated from the following equations:

Relative value = *V_s_* – *V_r_* (1)

CPA value = *V_r_’* – *V_r_* (2)

Finally, the electrodes were rinsed with a cleaning solution (100 mM KCl, 10 mM KOH, 30 vol% ethanol) and another standard solution. The measurement procedure was performed five times for each sample. The averages and standard deviations were calculated using the results excluding those for the first measurement.

### 2.4. Evaluation of Surface Conditions from Surface Zeta Potential and Contact Angle

The surface zeta potential and contact angle were measured to evaluate the change in the surface conditions due to the preconditioning process. First, the zeta potential of the lipid polymer membrane surface was measured by the streaming potential method using a SurPASS analyzer (Anton Paar GmbH, Graz, Austria) with an adjustable gap cell accessory. The solution used for the measurement was 1 mM KCl_(aq)_. A small amount of 100 mM KOH was added to the solution to control the pH to about 7 because the pH of the fresh KCl solution was about 5.8. The measurements were carried out while the solution was titrated with a small amount of 100 mM HCl. The measurements were conducted four times to obtain each data point. Second, the contact angle of the lipid/polymer membrane surface was measured by a DM500 contact angle meter (Kyowa Interface Science Co., Ltd., Saitama, Japan). The measurement was performed with a 2 μL water droplet. The measurements were conducted three times to obtain each data point.

### 2.5. Analysis of Substances on Lipid/Polymer Membrane Surface by FTIR-RAS

FTIR is one of the most convenient methods for the analysis of organic compounds. However, it is difficult to analyze thin film samples by FTIR, even if the attenuated total reflection (ATR) method is used, because the penetration depth of infrared light is about 500 nm using a germanium prism (or about 1000 nm using a diamond prism, which is commonly used). Therefore, we analyzed the organic substances on the surface of the lipid/polymer membrane by FTIR with reflection absorption spectroscopy (RAS) after the organic substances had been transferred to a gold surface.

The RAS method is used for the analysis of organic thin films on metal substrates. RAS is normally not able to analyze organic film surfaces (the substrate must be a flat metal). Therefore, after the substances on the lipid/polymer surface were transferred to a thin gold film by the following steps, we analyzed the organic layer on the gold film by FTIR-RAS. First, a thin gold film on a glass substrate was cleaned by piranha solution (H_2_SO_4_:H_2_O_2 (aq)_ = 4:1, 120 °C, 10 min) during which the surface of the gold film became clean and hydrophilic. Next, the surface of the lipid/polymer membrane was attached to the gold surface. Then, the lipid/polymer membrane was pressed from its rear side using a spatula. Next, the lipid/polymer membrane was lifted off from the gold surface. Finally, we analyzed the substances adsorbed on the gold surface using an FTIR system (Frontier Gold FTIR, PerkinElmer, Waltham, MA, USA) RAS with an Advanced Grazing Angle accessory including a polarizer (PIKE Technologies, Fitchburg, WI, USA) using an MCT detector.

### 2.6. Chemical Analyses of Surface of Lipid/Polymer Membrane

We used two methods for the analysis of organic substances on the surface in addition to FTIR-RAS. One of them was x-ray photoelectron spectroscopy (XPS), which is often used for surface analysis. For the analysis of organic substances, it is possible to recognize some chemical substances from the chemical shifts of nitrogen, oxygen and other elements. The analysis is highly sensitive within the surface region (within 2 nm). We evaluated the surface of the lipid/polymer membrane using an ESCA5800 system (ULVAC-PHI, Inc., Kanagawa, Japan) with a neutralizer. However, there were two problems in our XPS measurements. One of them was the possibility that some substances disappear by evaporation because the measurements were performed under ultrahigh vacuum (below 1 × 10^−9^ torr). Actually, the measurements were carried out after the lipid/polymer membrane samples had been stored under high vacuum (about 1 × 10^−6^ torr) for 2–3 weeks because it was impossible to measure the fresh membrane samples owing the vacuum. This suggests that some substances with low boiling points evaporated from the samples (for example, NPOE is a liquid at room temperature, whose boiling point is about 200 °C.). The other problem was contamination. It is difficult to distinguish target compounds and contaminants because it is difficult to identify a substance from information on the atomic and chemical shifts if not all the substances on the surface are known. We carried out gas cluster ion beam (GCIB) time of flight (TOF) secondary ion mass spectrometry (SIMS) with a cooled sample stage to solve these problems.

GCIB-TOF-SIMS is a method of analysis used to determine the depth profile of substances in an organic material. The analysis is performed by TOF-SIMS while etching the surface with an argon GCIB. The merit of etching with a GCIB is less damage to the substances than etching with an argon ion beam. If the etching is performed with an argon ion beam, it is difficult to identify the compounds because fragments of the compounds are generated during the etching. We evaluated the depth profile of substances in the lipid/polymer membrane starting from the surface, which was carried out by Toray Research Center (Tokyo, Japan) using a TOF.SIMS 5 (ION-TOF GmbH, Münster, Germany) instrument. It was possible to distinguish the target compounds and contaminants because the substances could be partially identified from the mass spectrum data. In addition, the effect of evaporation was reduced by cooling the samples to below −140 °C. In fact, it was possible to measure the fresh membrane samples without storing samples in a vacuum. However, there were two problems in our GCIB-TOF-SIMS measurement. One of them was that it was impossible to compare the concentrations of substances because the normalization of data could not be carried out owing to the lack of a standard sample. Such normalization is required to determine the absolute concentrations of the substances because the sensitivity of the mass detector is considerably different for each compounds. The other problem is that impurities in the materials affect the result because the mass detector has high sensitivity. In summary, to reveal the chemical substances on the lipid/polymer surface, we carried out XPS measurements with GCIB-TOF-SIMS measurement to support the XPS data.

## 3. Results and Discussion

### 3.1. Effect of Preconditioning of Lipid/Polymer Membrane

**Figure 1:**
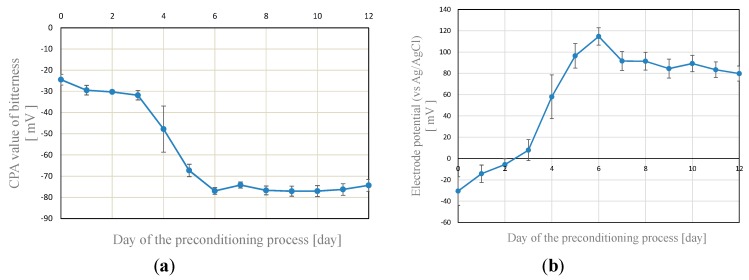
The result of measurements for bitterness sample (0.01 vol% iso-α-acid) depending on the preconditioning time. The five measurements were carried out at each day. The error bar is standard deviation of the data. (**a**) CPA value; (**b**) The electrode potential (*vs*. Ag/AgCl at room temperature) when the electrode was immersed in the standard solution.

The CPA value of the fresh lipid/polymer membrane was low. However, it became high when the membrane was immersed in MSG solution (30 mM in standard solution) for 4–5 days. The immersion process is called “MSG preconditioning”. Figure 1 shows the result of preconditioning. The five measurements for a bitterness sample (0.01 vol% iso-a-acid in standard solution) were carried out in one day. After the measurements, the lipid/polymer membrane was immersed in the MSG solution until the next day. The measurements were carried out for 13 days (0–12 days: total number of measurements = 65).

First, the CPA value (shown in Figure 1a) is low until about 3 days. After that, it starts to change then becomes stable at about -80 mV. The electrode potential, which is *V_r_* in Equations (1) and (2), is shown in Figure 1b. The transient behavior of the electrode potential is similar to that of the CPA value. Several similar experiments were carried out using other samples. Although the transients of the results were slightly different from each other, the stable CPA value and electrode potential were about −80 and +100 mV, respectively. This CPA value agrees with previously reported values [6]. In summary, the electrical property of the lipid/polymer membrane for bitterness sensing was changed by immersion (preconditioning) in MSG solution.

### 3.2. Surface Conditions of Lipid/Polymer Membrane

**Figure 2:**
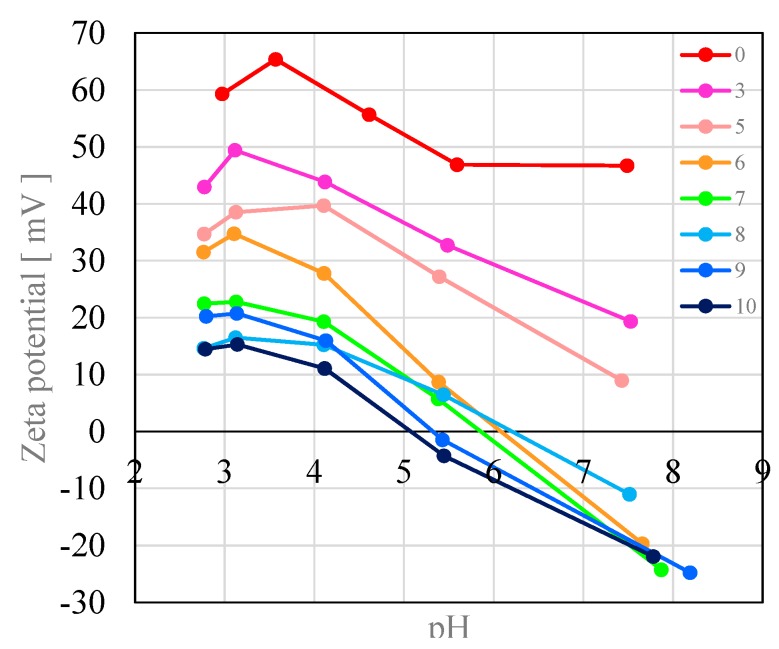
The dependence of the surface zeta potential during preconditioning. For example, the red graph is data of the membrane without the preconditioning. The dark blue graph is data of the membrane with the preconditioning for 10 days.

The electrode potential was affected by the preconditioning process. Figure 2 shows the change in the surface zeta potential with the progress of preconditioning. The potential became increasingly negative with increasing preconditioning time. This result suggests that the MSG molecules are adsorbed on the membrane surface because the molecules are negatively charged at pH higher than 3.22, which is the isoelectric point of MSG.

**Figure 3:**
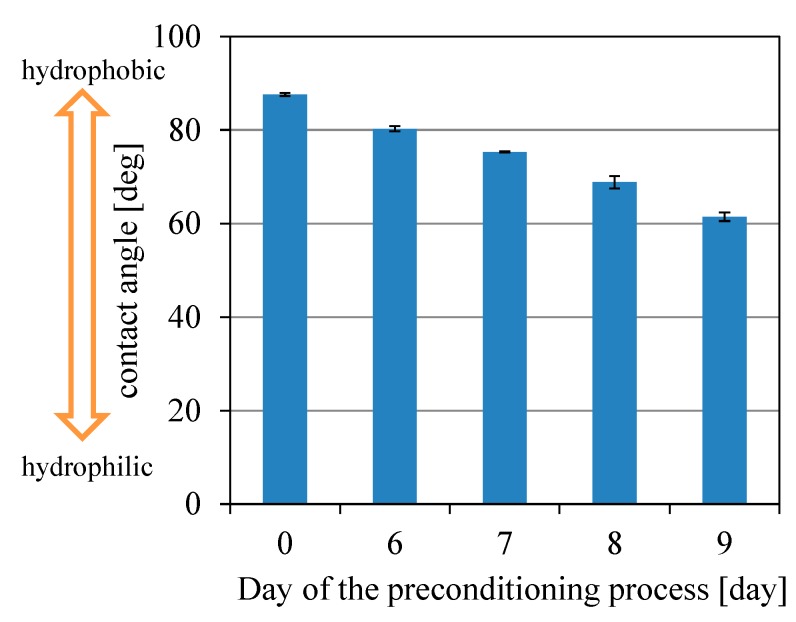
The contact angle of a water droplet with the lipid/polymer membrane.

The contact angle was measured to confirm the result of the surface zeta potential measurements during preconditioning process. Figure 3 shows the result. The contact angle decreased during preconditioning. This means that the surface became hydrophilic as a result of preconditioning. This result is consistent with that for the surface zeta potential because the surface becomes hydrophilic if the MSG molecules are adsorbed on the surface. In conclusion, the surface of the lipid/polymer membrane was negatively charged by the preconditioning and became hydrophilic.

### 3.3. FTIR-RAS Analysis of Substances on Lipid/Polymer Membrane Surface

**Figure 4:**
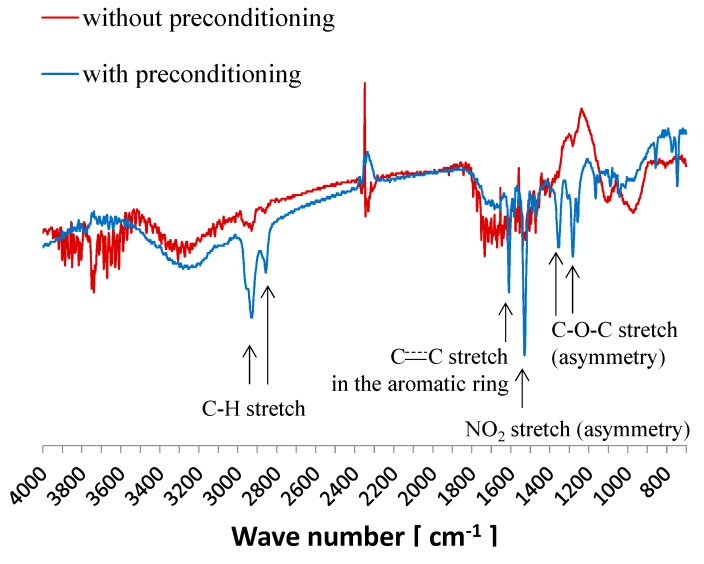
FTIR-RAS with transfer method for samples with and without the preconditioning for 7 days.

**Figure 5:**
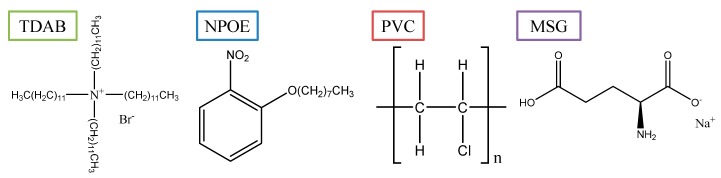
The chemical structures of TDAB, NPOE, PVC and MSG.

We found that the surface was changed by the preconditioning as reported in the previous section. Chemical analyses of the surface were carried out to reveal the reason for the change. First, FTIR-RAS by the transfer method was carried out in accordance with the procedure in Section 2.4. Figure 4 shows the results for samples with and without preconditioning for 7 days. Several peaks appear for the sample with the preconditioning, and that do not appear for the sample without the preconditioning. The peaks at 2860 and 2920 cm^−1^ result from C-H stretching, the peak at 1610 cm^−1^ results from C=C stretching in the aromatic ring, the peak at 1530 cm^−1^ results from NO_2_ asymmetric stretching, and the peaks at 1350 and 1280 cm^−1^ result from C–O–C asymmetric stretching. The chemical structure of the substance as below contained in the lipid/polymer membrane is shown in Figure 5. The above peaks suggest the chemical structure of NPOE. However, it would be difficult to recognize TDAB by FTIR even if it exists on the surface because the characteristic peaks of TDAB with high strength do not appear in IR data except for the peak corresponding to C–H stretching (we obtained IR data for the TDAB used in this study by FTIR-ATR in another experiment). In summary, NPOE is exuded onto the surface during the preconditioning.

### 3.4. Chemical Analysis of Surface of Lipid/Polymer Membrane by XPS

**Figure 6:**
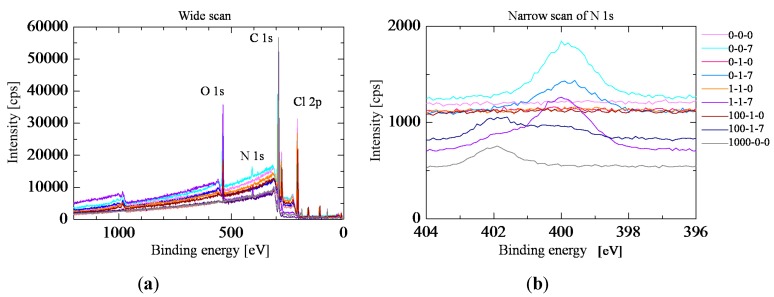
The result of XPS measurement. The upper graph is the result of wide scan. The lower graph is the result of narrow scan for signal of nitrogen 1s. The red lines are the results of samples without preconditioning. The blue lines are the result of samples with preconditioning. (**a**) Wide scan; (**b**) Narrow scan of Nitrogen 1s.

The analysis of samples with various NPOE and TDAB concentrations with and without preconditioning for 7 days was carried out by XPS. Table 1 shows the conditions of the samples. The samples that were analyzed by FTIR or other methods were samples 1-1-0 (without preconditioning) and 1-1-7 (with preconditioning). Sample 1-1-7 is used as a bitterness sensor. Figure 6 shows the result of the XPS measurements. First, we discuss the samples except for sample 1000-0-0 to simplify the explanation. As shown in the lower graph, the samples not subjected to preconditioning have no peak, whereas the samples subjected to preconditioning have a peak at 402 or 400 eV. This means that TDAB or NPOE was concentrated on the membrane surface by the preconditioning or that MSG was adsorbed on the surface by the preconditioning because TDAB, NPOE and MSG have a nitrogen atom. Next, sample 0-0-7, which has no TDAB or NPOE and was immersed in MSG solution, has a peak at 400 eV. Thus, the peak at 400 eV originates from the MSG. Next, the intensity of the peak at 402 eV for samples 0-0-7, 0-1-7, 1-1-7 and 100-1-7 depends on the amount of TDAB. In addition, sample 1000-0-0, which has only TDAB, has a peak at 402 eV. Therefore, the peak at 402 eV originates from TDAB. Moreover, the intensities of the peak at 402 eV for samples 1-1-7 and 100-1-7 are higher than those of samples 1-1-0 and 100-1-0, which were not subjected to preconditioning. This means that the TDAB on the surface was concentrated by the preconditioning. In conclusion, the preconditioning process concentrated the TDAB on the lipid/polymer surface and caused the adsorption of MSG on the surface.

**Table 1:** The samples for XPS measurement. The first and second numbers are the amount of TDAB and NPOE, respectively. The last number is the preconditioning time. For example, sample 100-1-7 means that the amount of TDAB is 100 times of sample 1-1-7, which is used as a bitterness sensor.

Sample Name	TDAB (times)	NPOE (times)	Preconditioning Time [ day ]
0-0-0	0x	0x	0
0-0-7	0x	0x	7
0-1-0	0x	1x	0
0-1-7	0x	1x	7
1-1-0	1x	1x	0
1-1-7	1x	1x	7
100-1-0	100x	1x	0
100-1-7	100x	1x	7
1000-0-0	1000x	0x	0

### 3.5. Analysis of Depth Profile of Substances in Lipid/Polymer Membrane

**Figure 7:**
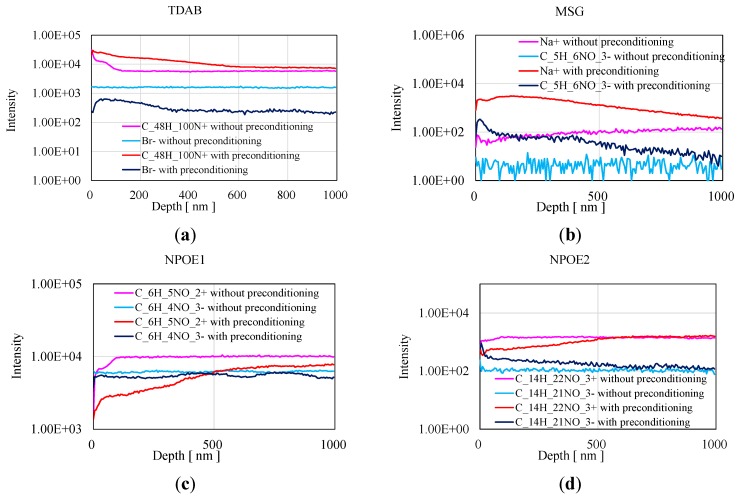
The result of GCIB-TOF-SIMS measurement. Four lines indicate the data of samples with and without the preconditioning using positive and negative ion. ATTENTION: The intensities cannot compare with each other samples because of no normalization. (**a**) ^69^°C_48_H_100_N^+^ and ^81^Br**^−^** of TDAB; (**b**) ^23^Na^+^ and ^128^C_5_H_6_NO_3_**^−^** of MSG; (**c**) ^123^C_6_H_5_NO_2_^+^ and ^138^C_6_H_4_NO_3_**^−^** of NPOE; (**d**) ^252^C_14_H_22_NO_3_^+^ and ^251^C_14_H_21_NO_3_**^−^** of NPOE.

The depth profile of the concentration of substances in the lipid/polymer membrane was measured by GCIB-TOF-SIMS to confirm the result of XPS. Figure 7 shows the result of GCIB-TOF-SIMS, which was carried out starting from the surface. The concentrations of TDAB and MSG in the surface region are higher than those in the deep region after the preconditioning. TDAB was concentrated in the surface region, enabling it to be trapped at the interface between the MSG aqueous solution and the membrane via the quaternary amino group of TDAB, which is hydrophilic. MSG diffused from the solution into the membrane. This result supports the result of XPS analysis. In addition, we found that the concentrations of TDAB and MSG after preconditioning are higher up to a depth of 1000 nm, although we expected that they would have only been concentrated at the surface. However, we could not explain the data for NPOE because the results for positive and negative ions were different. There might be problem in measuring NPOE by GCIB-TOF-SIMS because the result of FTIR-RAS in Figure 4 suggests that the amount of NPOE increased on the surface. In conclusion, the concentrations of TDAB and MSG were higher in the surface region than in the bulk region.

## 4. Conclusions

We analyzed the surface of a lipid/polymer membrane for bitterness sensing to determine the effect of the MSG preconditioning process. The purpose of this study was to explain why it becomes possible to measure bitterness by CPA using the lipid/polymer membrane after the preconditioning process. We evaluated the surface conditions using electrical and chemical methods. First, the contact angle and the surface zeta potential were measured as electrical methods because those have a relationship with the response of the taste sensor. The results indicated that the surface became hydrophilic and was negatively charged by the preconditioning. Next, FTIR-RAS, XPS and GCIB-TOF-SIMS were carried out as chemical methods to identify substances on the surface. Their results indicated that the adsorption of MSG on the surface, and that TDAB was concentrated on the surface. However, the results did not support that of FTIR-RAS measurement, which showed that NPOE was exuded on the surface. In this study, we revealed the change in the surface structure caused by MSG preconditioning. In our future work, we will clarify the relationship between the mechanism of CPA measurement and the change in the surface structure.
